# A simple high-throughput approach identifies actionable drug sensitivities in patient-derived tumor organoids

**DOI:** 10.1038/s42003-019-0305-x

**Published:** 2019-02-25

**Authors:** Nhan Phan, Jenny J. Hong, Bobby Tofig, Matthew Mapua, David Elashoff, Neda A. Moatamed, Jin Huang, Sanaz Memarzadeh, Robert Damoiseaux, Alice Soragni

**Affiliations:** 1 Division of Hematology-Oncology, David Geffen School of Medicine, University of California, Los Angeles, CA 90095 USA; 2 Molecular Screening Shared Resource, California NanoSystems Institute, University of California, Los Angeles, CA 90095 USA; 3 Department of Biostatistics, David Geffen School of Medicine, University of California, Los Angeles, CA 90095 USA; 4 Department of Pathology, David Geffen School of Medicine, University of California, Los Angeles, CA 90095 USA; 5 Department of Obstetrics and Gynecology, David Geffen School of Medicine, University of California, Los Angeles, CA 90095 USA; 6 Eli and Edythe Broad Center of Regenerative Medicine and Stem Cell Research, University of California, Los Angeles, CA 90095 USA; 7 The VA Greater Los Angeles Health Care System, Los Angeles, CA 90073 USA; 8 Department of Biological Chemistry, University of California, Los Angeles, CA 90095 USA; 9 Molecular Biology Institute, University of California, Los Angeles, CA 90095 USA; 10 Department of Molecular and Medicinal Pharmacology, David Geffen School of Medicine, University of California, Los Angeles, CA 90095 USA; 11 Present Address: Laboratory of Stem Cell Research and Application, University of Science, Vietnam National University, HCM City, Vietnam

## Abstract

Tumor organoids maintain cell–cell interactions, heterogeneity, microenvironment, and drug response of the sample they originate from. Thus, there is increasing interest in developing tumor organoid models for drug development and personalized medicine applications. Although organoids are in principle amenable to high-throughput screenings, progress has been hampered by technical constraints and extensive manipulations required by current methods. Here we introduce a miniaturized method that uses a simplified geometry by seeding cells around the rim of the wells (mini-rings). This allows high-throughput screenings in a format compatible with automation as shown using four patient-derived tumor organoids established from two ovarian and one peritoneal high-grade serous carcinomas and one carcinosarcoma of the ovary. Using our automated screening platform, we identified personalized responses by measuring viability, number, and size of organoids after exposure to 240 kinase inhibitors. Results are available within a week from surgery, a timeline compatible with therapeutic decision-making.

## Abstract (web summary)

Nhan Phan et al. present a high-throughput approach to screen tumor organoids by seeding cells in mini-rings. They apply their method to cell lines and patient-derived tumor organoids representing four different cancers, and identified personalized responses for each organoid within a clinically relevant timeline.

## Introduction

Cancer therapy is rapidly progressing toward individualized regimens not based on the organ of origin, but rather on the molecular characteristics of tumors. Next-generation sequencing is typically regarded as the key to access this potentially actionable molecular information^1,2^. However, recent studies showed how only a small number of cancers can be singled out and targeted with this approach, in part because very few gene alteration–drug pairs are unequivocally established and few accurate predictive biomarkers are available^3–7^. Thus, functional precision therapy approaches where the primary tumor tissue is directly exposed to drugs, to determine which may be efficacious, have the potential to boost personalized medicine efforts and influence clinical decisions^3,4^. Establishing patient-derived xenografts (PDXs) is a costly and time-consuming option that only allows to screen very few potential drugs. Conversely, ex vivo three-dimensional (3D) tumor spheroids or organoids derived from primary cancers can be easily established and potentially scaled to screen hundreds to thousands of different conditions.

3D cancer models have been consistently shown to faithfully recapitulate features of the tumor of origin in terms of cell differentiation, heterogeneity, histoarchitecture, and clinical drug response^4,8–16^. Various methods to set up tumor spheroids or organoids have been proposed, including using low-attachment U-bottom plates, feeding layers, or various biological and artificial matrices^9,12,13,16–23^. Methods using low-attachment U-bottom plates ideally only carry one organoid per well and have limited automation and final assay capabilities^19–21^. In addition, not all cells are capable of forming organized 3D structures with this method. Approaches that include a bio-matrix, such as Matrigel, have the potential to offer a scalable alternative in which cancer cells thrive^9,14,24,25^. However, most methods proposed so far rely on thick volumes of matrix, which is not cost-effective, potentially hard for drugs to efficiently penetrate, and difficult to dissolve fully at the end of the experiment^4,24^. In other applications, organoids are first formed and then transferred to different plates for drug treatment or final readout, which can result in the tumor spheres sticking to plastic or breaking^14,25^. In addition, some assays require to disrupt the organoids to single-cell suspensions at the end of the experiment^17,23^. All of these manipulations introduce large variability, limiting applicability in screening efforts^12^.

To overcome these limitations, we introduce a facile assay system to screen 3D tumor organoids that takes advantage of a specific geometry. Our miniaturized ring methodology does not require functionalized plates. Organoids are assayed in the same plate where they are seeded, with no need for sample transfer at any stage or dissociation of the pre-formed tumor organoids to a single-cell suspension. Here we show that the mini-ring approach is simple, robust, requires few cells, and can be easily automated for high-throughput applications. Using this method, we were able to rapidly identify clinically actionable drug sensitivities for several ovarian cancers and high-grade serous tumors by testing two different drug concentrations and a library of 240 protein kinase inhibitor compounds.

## Results

### Establishment of 3D tumor models in ring format

**Fig. 1:**
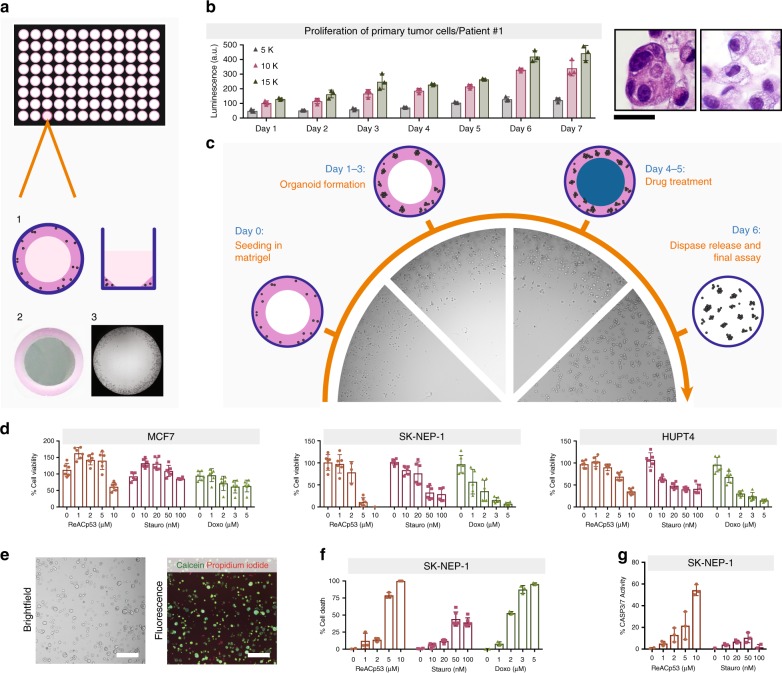
The mini-ring method for 3D tumor cell biology. **a** Schematics of the mini-ring setup. Cells are plated to form a solid thin ring as depicted in 1 and photographed in 2. The picture in 3 acquired with a cell imager shows tumor organoids growing at the periphery of the well as desired, with no invasion of the center. **b** Proliferation of primary tumor cells as measured by ATP release. Different seeding densities were tested and compared. This clinical sample grew and maintained the heterogeneity and histology of the original ovarian tumor, which had a high-grade serous carcinoma component (H&E left picture) and a clear cell component (H&E right picture). Scale bar, 20 µm. **c** Schematic of the drug-treatment experiments performed in the mini-ring setting. The pictures are representative images as acquired on different days using a Celigo cell imager. **d**–**g** Assays to monitor drug response of cell lines using the mini-ring configuration. Three drugs (ReACp53, Staurosporine, and Doxorubicin) were tested at five concentrations in triplicates for all cell lines. **d** ATP release assay (CellTiter-Glo 3D) readout. **e**,**f** Calcein/PI readout. **e** Representative image showing staining of MCF7 cells with the dyes and segmentation to quantify the different populations (live / dead). Scale bar, 400 nm. **f** Quantification of Calcein/PI assay for three-drug assay. **g** Quantification of cleaved caspase 3/7 assay. Doxorubicin was omitted due to its fluorescence overlapping with the caspase signal. For all graphs, symbols are individual replicates, bars represent the average, and error bars show SD

To rapidly screen organoids, we first established a miniaturized system that allows the setup of hundreds of wells and perform assays with minimal manipulation. We adapted the geometry used to plate tumor cells in Matrigel, to generate mini-rings around the rim of the wells. This is attained by plating single-cell suspensions obtained from a cell line or a surgical specimen pre-mixed with cold Matrigel (3:4 ratio) in a ring shape around the rim in 96-well plates (Fig. 1a). Rings can be established using a single-well or multichannel pipette. Use of a robotic system or automated 96-well pipettor is theoretically feasible as long as temperature and plate positioning can be effectively controlled. The combination of small volume plated (10 µl) and surface tension holds the cells in place until the Matrigel solidifies upon incubation at 37 °C and prevents two-dimensional (2D) growth at the center of the wells. The ring configuration allows for media addition and removal so that changes of conditions or treatment addition to be easily performed by pipetting directly in the center of the well, preventing any disruption of the gel. Cancer cell lines grown in mini-ring format give rise to organized tumor organoids that recapitulate features of the original histology (Supplementary Fig. 1 and Supplementary Table 1).

Similarly, we can routinely establish patient-derived tumor organoids (PDTOs) using the same geometry. As an example, Patient #1 was diagnosed with a high-grade mixed type carcinoma with both a high-grade serous component as well as a clear cell component (Supplementary Table 1 and Supplementary Fig. 2a). Cancer cells isolated from Patient #1 grown in our ring system show two distinct cytomorphologies: one group of cells have clear cytoplasm and cuboidal appearance, whereas the second group of cells organize in clusters in a columnar manner and have dense cytoplasm (Supplementary Fig. 2a). These morphologies are compatible with the two different histologies found in the original tumor, clear cell, and high-grade serous carcinoma (Supplementary Fig. 2a).

p53 is a defining marker of serous ovarian cancer, but is rarely expressed by clear cell ovarian tumors^26^. Both the tumor organoids and the primary cancer cells show similar p53 staining patters, with populations of p53-positive and p53-negative cells (Supplementary Fig. 2b,c). Thus, patient samples obtained at the time of surgery can proliferate in our system and maintain the heterogeneity of the original tumor as expected (Fig. 1b and Supplementary Fig. 2).

### Assay optimization

Next we optimized treatment protocols and readouts for the mini-ring approach. Our standardized paradigm includes: seeding cells on day 0, establishing organoids for 2–3 days followed by two consecutive daily drug treatments, each performed by complete medium change (Fig. 1c). To demonstrate feasibility, we performed small-scale screenings testing three drugs at five different concentrations in triplicates, ReACp53^17^, Staurosporine^27^, and Doxorubicin (Fig. 1d–g, Supplementary Fig. 3–5). We optimized different readouts to adapt the method to specific research questions or instrument availability. After seeding cells in standard white plates, we performed a luminescence-based ATP assay to obtain a metabolic readout of cell status, calculate EC_50_, and identify cell-specific sensitivities (Fig. 1, Supplementary Figs. 3–4). Results show how the Matrigel in the mini-ring setup is thin enough to allow penetration not only of small molecules but also of higher molecular weight biologics such as peptides^17^. EC_50_s ranged between 2.5 µM (MDA-MB-468) and 10 µM (MCF7) for ReACp53, between 100 nM (MCF7) and 800 nM (PANC 03.27) for Staurosporine, and between 0.9 µM (SK-NEP) and 12 µM (MCF7) for Doxorubicin. Our measurements are in line with the Doxorubicin resistance of MCF7 cells grown in Matrigel in 3D that has been previously reported^28^.

We performed two consecutive treatments, which allows the drugs to not only penetrate the gel but also to reach organoids that may be bulky^17^. However, the assay is flexible and can be easily adapted to single treatments followed by longer incubations, multiple consecutive recurring treatments, multi-drug combinations, or other screening strategies (Supplementary Fig. 4).

We also implemented assays to quantify drug response by measuring cell viability after staining of live organoids with specific dyes followed by imaging. We optimized a calcein-release assay coupled to propidium iodide (PI) staining as well as a caspase 3/7 cleavage assay that can be readily performed after seeding the cells in standard black plates (Fig. 1e–g and Supplementary Fig. 5). For all assays, tumor organoids are stained following dispase release. After a 40 min incubation, organoids are imaged and pictures are segmented and quantified (Fig. 1e–g and Supplementary Fig. 5). All the assays are performed within the same well in which spheroids are seeded. Although the various assays we introduce are testing different aspects of cell viability and measure distinct biological events, results were mostly concordant across the methods for the three drugs tested (Fig. 1 and Supplementary Figs. 3 and 5).

### Comparison of mini-ring method with traditional drop seeding

**Fig. 2:**
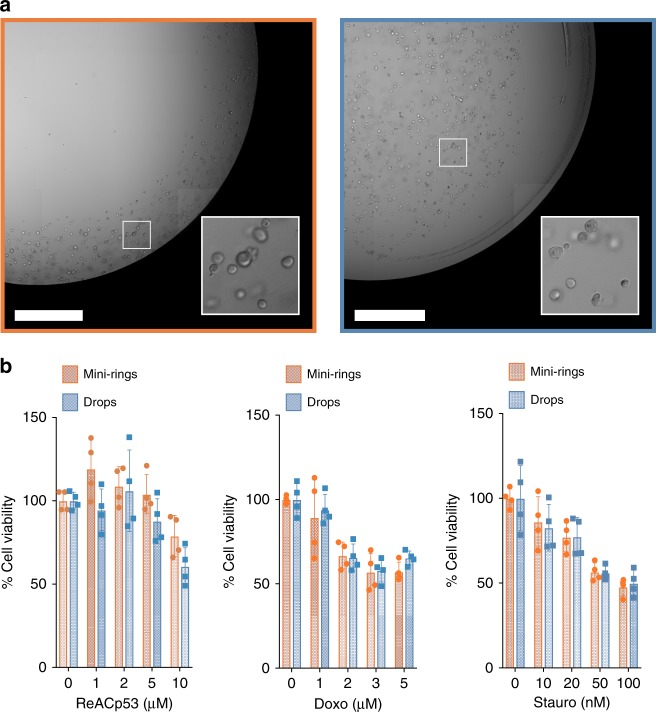
Comparison of different seeding procedures. **a** Bright-field images of rings and drops of MCF7 cells in Cultrex BME. Scale bar, 1 mm. **b** ATP assays showing identical sensitivities of mini-rings and drops to ReACp53, Staurosporine, and Doxorubicin tested at five concentrations in duplicates. Two independent experiments performed, all points shown. Bars represent the average, error bars show SD

To confirm that 3D models established in mini-rings behave as those formed using traditional drop seeding methods, we directly compared the two techniques (Fig. 2). For this experiment, we seeded 5000 MCF7 cells/well either as drops or mini-rings and tested three drugs, ReACp53, Staurosporine, and Doxorubicin, in duplicates as described above. Results show that appearance of MCF7 3D spheroids (Fig. 2a) and drug sensitivities as measured by ATP assays (Fig. 2b) were undistinguishable when comparing mini-rings and drops. However, drops required individual manual aspiration and media addition, which resulted in longer processing times as no automation could be implemented.

Many other proteinaceous matrices are commercially available beside Matrigel. To confirm that other supports can be used for mini-rings, we used Cultrex BME in this experiment instead of Matrigel. Cells could be seeded as mini-rings and performance of Cultrex BME mirrored that of Matrigel (see Fig. 1d vs. Fig. 2b). In summary, different supports can be used to establish 3D models in mini-ring format and we observe no effect of mini-rings in terms of growth and drug treatment when comparing these with traditional seeding approaches.

### Identification of actionable drug responses in PDTOs

**Fig. 3:**
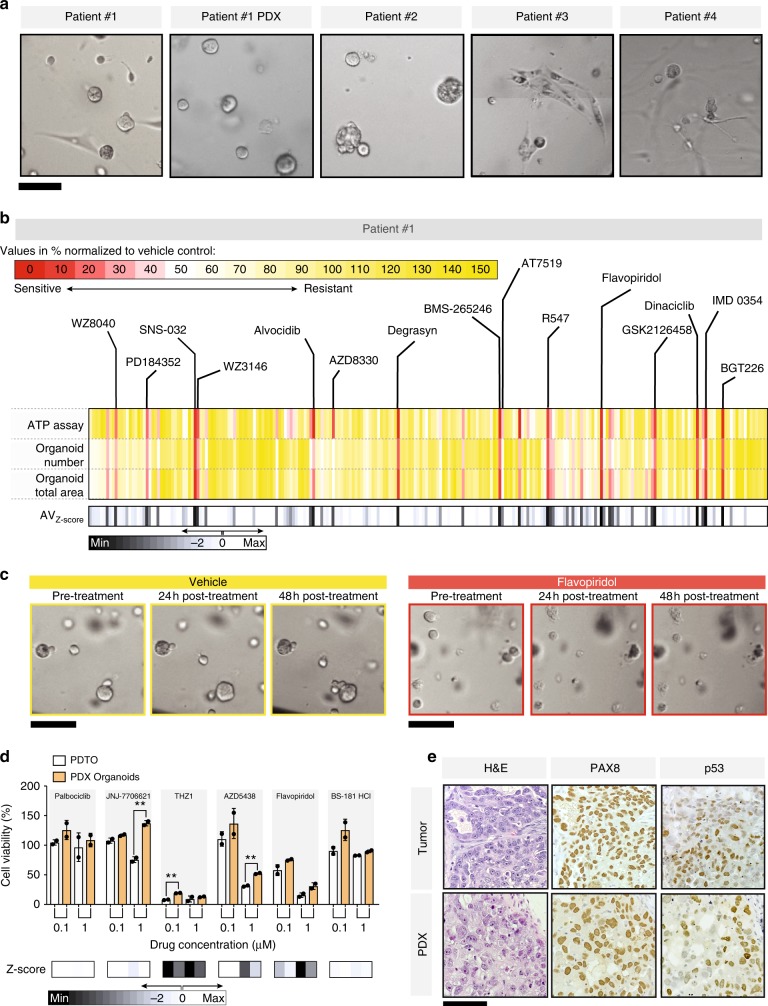
Mini-ring approach to unveil drug response patterns in PDTOs. **a** Morphology of all PDTOs established in this study as visualized by bright-field microscopy. Morphology and 3D organization of the samples is highly variable. For instance, some of Patient #3 cells are arranged in fascicles within the Matrigel, likely representing the sarcomatous component of the tumor. Scale bar, 100 µm. **b** Results of kinase screening experiment for Patient #1 PDTOs. Three readouts were used for this assay: ATP quantification as measured by CellTiter-Glo 3D and organoid number or size quantification evaluated by bright-field imaging. Bright-field images were segmented and quantified using the Celigo S Imaging Cell Cytometer Software. Both organoid number and total area were evaluated for their ability to capture response to drugs. In this plot, each vertical line is one drug, all 240 tested are shown. Values are normalized to the respective vehicle controls for each method and expressed as %. Average_Z-score_ calculated as reported in Methods. **c** A representative image of the effects of the indicated drug treatments as visualized by the Celigo cell imager. Scale bar, 100 µm. **d** Small-scale kinase assay on Patient #1 primary PDTOs and PDX-derived cells. ATP readout. Four molecules not present in the primary screening were tested. Flavopiridol and BS-181 HCl are included as positive and negative control, respectively. *t*-test, **p < 0.01. **e** Comparison of the histology of the primary tumor with the established PDX. Scale bar: 100 µm

**Fig. 4:**
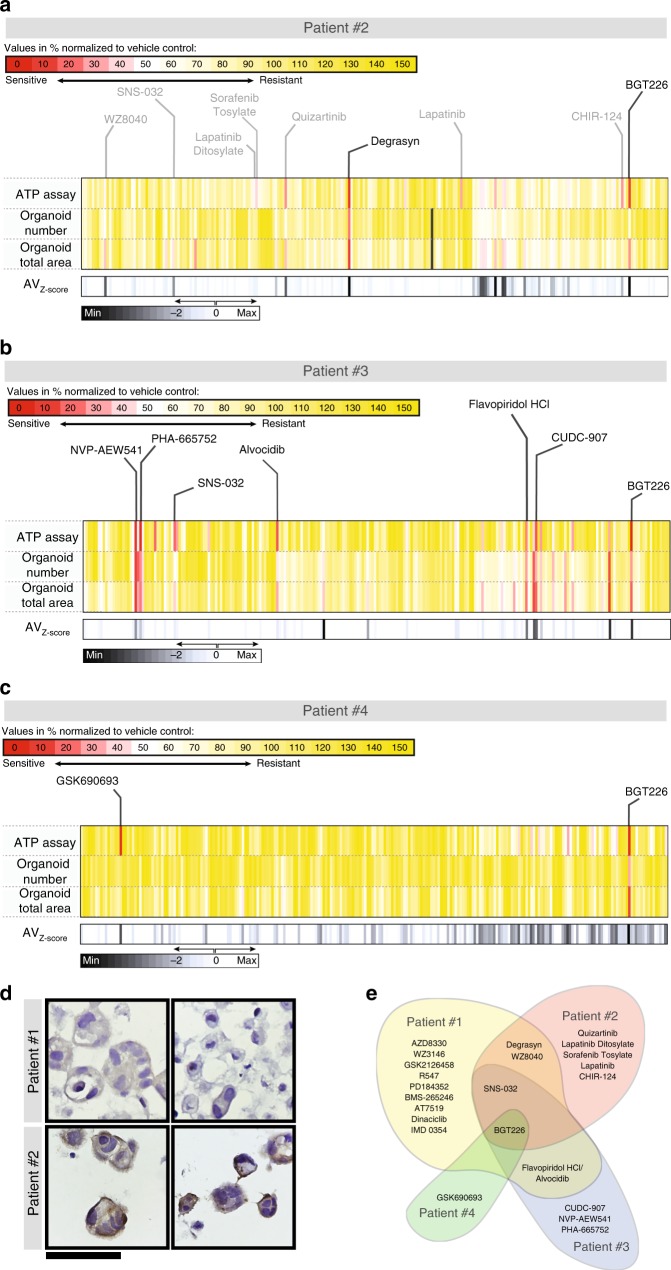
Individualized response of PDTOs to tyrosine kinase inhibitors. **a**–**c** Results of kinase screening experiment on Patients #2–4 organoids. Each vertical line represents one of 241 tested drugs. Values are normalized to the respective vehicle controls (DMSO) for each method and expressed as %. **d** Expression of the multi-drug efflux protein ABCB1 in PDTOs as visualized by IHC. Patient #2 expresses very high levels of the ABC transporter. Scale bar: 60 µm. **e** Diagram illustrating limited overlap between the detected patterns of response identified through the mini-ring assay for all patients

A rapid functional assay to determine drug sensitivities of primary specimens can offer actionable information to help tailoring therapy to individual cancer patients^3^. We tested suitability of our approach to rapidly and effectively identify drug susceptibilities of three ovarian cancer samples and one high-grade serous peritoneal cancer specimen obtained from the operating room (Supplementary Table 1; Figs. 3 and 4). In all cases, ascites or tumor samples were processed and then plated as mini-rings (see Methods). To maximize the amount of information extracted from irreplaceable clinical samples, we investigated the possibility to concurrently perform multiple assays on the same plate. To do so, we first optimized the initial seeding cell number (5000 cells/well) to couple an ATP metabolic assay to 3D tumor count and total organoid area measurements. This seeding density yields a low-enough number of organoids to facilitate size distribution analysis but sufficient ATP signal to be within the dynamic range of the CaspaseGlo 3D assay. Careful consideration should be given as to whether the number of seeding cells can accurately recapitulate composition and heterogeneity of the tumor of origin. Cancer cell concentration can be reduced or augmented in our system depending on the characteristics of the tumor (Fig. 1b).

For each patient sample, we seeded six 96-well plates and tested 240 protein kinase inhibitors FDA-approved or in clinical development. We tested each drug at two different concentrations (120 nM and 1 µM), for a total of 480 different conditions tested. Differently from established cancer cell lines, the number of cells obtained from surgical specimens can be limiting. As such, we opted for a two-dose focused screening, a common approach to identify potential hits. Validation can then be performed using frozen aliquots of cells that we cryopreserve after tissue processing post surgery (Supplementary Fig. 6b). However, our method can be adapted to accommodate any number of different screening designs, including concentration series (Fig. 1d–g and Supplementary Fig. 3 and 5) or multiple drug combinatorial assays.

For PDTOs, we used the same experimental paradigm optimized using cell lines (Fig. 1c). All steps (media change, drug treatment) were automated and performed in < 2 min/plate using a Beckman Coulter Biomek FX integrated into a Thermo Spinnaker robotic system. At the end of each experiment, PDTOs are first imaged in bright-field mode for organoid count/size distribution analysis followed by an ATP assay performed on the same plates. The measurements yielded high-quality data that converged on several hits, highlighting the feasibility of our approach to identify potential leads (Figs. 3 and 4).

### Patient #1: high-grade mixed type carcinoma

Cells obtained from Patient #1 at the time of cytoreductive surgery were chemo-naive and the heterogeneous nature of this clear cell/high-grade serous tumor was recapitulated in the PDTOs (Table 1, Fig. 1b, and Supplementary Fig. 2). Despite aggressive debulking surgery and treatment with carboplatin and paclitaxel regimens, Patient #1 had persistent disease, never achieved complete remission, and overall survival from diagnosis was 11 months. Resistance to carboplatin was also observed in our high-throughput assay, with no significant reduction of viability observed at either 10 or 25 µM concentrations (Supplementary Fig. 6a). The organoids were however sensitive to ~6% of the protein kinase inhibitors tested (16/240), with sensitivity defined as residual cell viability ≤ 25% and average *Z*-score ≤ − 5 (Table 1, Supplementary Table 2, Supplementary Fig. 7a; see Methods for *Z*-score calculations). Patient #1’s tumor organoids responded to 58% of all cyclin-dependent kinase (CDK) inhibitors tested (7/12 total, 11 different compounds, and one, Flavopiridol, in two formulations). In particular, cells appeared highly sensitive to inhibitors hitting CDK1/2 in combination with CDK4/6 or CDK5/9 (Table 1, Fig. 3c, and Supplementary Table 3). Interestingly, CDK inhibitors have found limited applicability in ovarian cancer therapy so far^29^. Based on the profiles of the CDK inhibitors tested and on the response observed (Supplementary Table 3), we selected four untested molecules to assay. We anticipated that Patient #1 would not respond to Palbociclib (targeting CDK4/6) and THZ1 (CDK7), while expecting a response to JNJ-7706621 (CDK1/2/3/4/6) and AZD54338 (CDK1/2/9; Supplementary Table 3). BS-181 HCl and Flavopiridol were included as negative and positive control, respectively. Results show that organoids were not sensitive to JNJ-7706621 but had a strong response to THZ1 (Fig. 3d). Both THZ1 and BS-181 HCl specifically target CDK7. Nevertheless, Patient #1 PDTOs showed a strong response to the former but no response to the latter, which could be attributed to the different activity of the two as recently observed in breast cancer^30^. We detected elevated CDK7 protein expression in Patient #1 PDTOs (Supplementary Fig. 7b).

**Table 1:** List of molecules causing over 75% reduction in viability in PDTOs established from Patient #1’s tumor

	Drug	Target	AV_Z-score_	Cell Viability (%)
Patient #1	SNS-032	CDK	− 8.0	6.7
	Alvocidib	CDK	− 7.7	2.9
	AT7519	CDK	− 7.3	4.2
	BMS-265246	CDK	− 7.6	5.0
	Flavopiridol HCl	CDK	− 8.6	6.0
	R547	CDK	− 8.1	11.8
	Dinaciclib	CDK	− 8.3	8.1
	Degrasyn	DUB, Bcr-Abl	− 7.6	5.4
	WZ3146	EGFR	− 6.9	16.2
	WZ8040	EGFR	− 6.5	22.0
	IMD 0354	IKK	− 8.6	4.8
	PD184352	MEK	− 6.7	20.1
	AZD8330	MEK	− 6.1	15.5
	GSK2126458(Omipalisib)	PI3K, mTOR	− 7.9	11.6
	BGT226	PI3K, mTOR	− 8.5	5.0

We also attempted to validate the screening results in vivo by establishing PDXs injecting Patient #1 cells subcutaneously in NSG mice (500 K/mouse, 12 mice). However, only three mice developed PDXs over the course of 5 months. The xenografts resembled the histology of the primary tumor (Fig. 3e). To test whether the PDXs had a similar response to CDK inhibitors, we dissociated the PDX to single-cell suspension and generated organoids from one of them (Fig. 3a, d, e). The PDX-derived organoids showed an overall trend toward a reduction in sensitivity to CDKs when compared with the PDTOs. We observed a statistically significant decrease in response to 0.1 µM THZ1, and 1 µM JNJ-7706621 and AZD5438 (*p* < 0.01, Fig. 3d) in the PDX-derived organoid compared with the PDTOs. This is not unexpected, as human cancer cells grown in mice rapidly diverge from the tumor they were obtained from^31,32^.

### Patient #2: platinum-resistant high-grade serous ovarian carcinoma

Patient #2 was diagnosed with progressive, platinum-resistant high-grade serous ovarian cancer and was heavily pretreated before sample procurement (Supplementary Table 1). Patient #2 PDTOs were also platinum-resistant in our system (Supplementary Fig. 6a), with no reduction of viability observed upon treating the cells with either 10 or 25 µM carboplatin. The PDTOs showed a strong response (residual cell viability ≤ 25% and average *Z*-score ≤ − 5) to only 0.8% of all drugs tested (2/240, Fig. 4a, Table 2, and Supplementary Fig. 7a). We validated the results by performing a dose–response study (Supplementary Fig. 6b). We exposed patient #2 organoids to eight concentrations of the two hits identified in the screening, BGT226 and Degrasyn (0, 0.05, 0.1, 0.25, 0.5, 1, 5, and 10 µM), in duplicates. We used the same experimental setup as indicated above and the EC_50_s calculated using the ATP results from two independent experiments confirm Patient #2’s organoid sensitivity to low concentrations of the two drugs (Supplementary Fig. 6b).

**Table 2:** Drug leads causing over 75% cell death in PDTOs from Patient #2, #3, and #4

		Drug name	Target
Patient	#2	Quizartinib	Flt
		BGT226	PI3K, mTOR
		Degrasyn	DUB, Bcr-Abl
		Response comparable to Staurosporine*:	
		Lapatinib Ditosylate	EGFR, HER2
		Sorafenib Tosylate	VEGFR, PDGFR, Raf
		WZ8040	EGFR
		Lapatinib	EGFR, HER2
		CHIR-124	Chk
	#3	SNS-032	CDK
		Flavopiridol HCl	CDK
		Alvocidib	CDK
		BGT226	PI3K, mTOR
		CUDC-907	HDAC, PI3K
		CUDC-101	HDAC, EGFR, HER2
		NVP-AEW541	IGF-1R
		PHA-665752	c-Met
	#4	GSK690693	Akt
		BGT226	PI3K, mTOR

*Cell viability > 25% and ≤ (Staurosporine + 5%) at 1 µM

Patient #2 PDTOs showed only a moderate response to our positive control, Staurosporine, a pan-kinase inhibitor with very broad activity^27^. The lack of response to multiple therapies observed for Patient #2 led us to hypothesize that there could be overexpression of efflux membrane proteins. Indeed, the PDTOs showed a high level of expression of ABCB1 (Fig. 4d). High expression of the ATP-dependent detox protein ABCB1 is frequently found in chemoresistant ovarian cancer cells and recurrent ovarian cancer patients’ samples, and has been correlated with poor prognosis^33,34^.

We found a moderate response, comparable to the effect of Staurosporine (~40% residual cell viability), to EGFR/HER2 inhibitors including Lapatinib and WZ8040 (Table 2). We could detect high expression of EGFR at the plasma membrane of the tumor cells (Supplementary Fig. 7c), as is common for platinum-resistant ovarian cancer^35^.

### Patients #3: carcinosarcoma of the ovary

Patient #3 presented with a carcinosarcoma of the ovary, an extremely rare and aggressive ovarian tumor, which has not been fully characterized at the molecular level yet^36,37^ (Supplementary Table 1, Fig. 4b, Table 2, and Supplementary Fig. 7a). In our screening, the PDTOs established from this tumor responded to ~3% of all tested kinase inhibitors (7/240, residual cell viability ≤ 25%, and average *Z*-score ≤ − 1.5), including CDK inhibitors and phosphatidyl inositol 3-kinase (PI3K) inhibitors.

### Patient #4: high-grade peritoneal carcinoma

Patient #4 was diagnosed with a high-grade peritoneal tumor and showed a response to only 0.8% of all tested drugs (2/240, Supplementary Table 1, Fig. 4c, Table 3, and Supplementary Fig. 7a and 7d). The PDTOs showed a marked response to two drugs, one pan-Akt inhibitor (GSK690693) and a PI3K/mammalian target of rapamycin (mTOR) inhibitor (BGT226), with measured cell viability ≤ 25% and average *Z*-score ≤ − 5. However, different from Patient #2, Patient #4 PDTOs were sensitive to Staurosporine, with only 9 ± 1% residual viability after 2 days of treatment. Protein kinase C, which is the primary target of Staurosporine, is also a secondary target of GSK690693^38^.

Although only 2 inhibitors caused a 75% reduction in cell viability, 11 agents caused ≥ 50% cell death (*Z*-score ≤ − 5). Using this cutoff, we could identify six mTOR inhibitors including Omipalisib, Apitolisib, and Sapanisertib. These constitute 30% of all the mTOR inhibitors tested, pinpointing a potential vulnerability of this pathway.

## Discussion

We devised and optimized a facile high-throughput approach to establish and screen 3D models and tumor organoids generated from cell lines or clinical samples. We used our approach to functionally profile four tumors obtained from surgeries. We observed highly tumor-specific responses, with very little overlap among inhibitors that each clinical sample was sensitive to. Only one inhibitor, BGT226, showed activity in all tumors (Fig. 4e). A phase I basket trial of this PI3K/mTOR inhibitor showed moderate responses in unstratified patients^39^. PI3K/mTOR inhibitors are just one example of drugs for which a clear predictive marker is lacking, and patient with or without PI3K alterations have been shown to respond to PI3K inhibitors^39^. In fact, the absence of specific, unequivocal biomarkers predictive of response is a common limitation and challenge associated with the clinical implementation of many kinase inhibitors^40,41^. Our assay could bypass the lack of predictors of response and identify responsive tumors from a functional standpoint. Thus, patients may greatly benefit from functional PDTO testing, either to identify a suitable therapy or to facilitate patient selection for clinical trials^3,4,12,14,42^.

A recent study by Vlachogiannis et al.^4^ found that patient-derived organoids could accurately predict patient responses to therapy, with 100% sensitivity and 93% specificity. In our experiments, we could recapitulate the carboplatin resistance of patients ex vivo (Supplemnetary Fig. 6a). Interestingly, PDTOs exhibited differential responses to different molecules targeting the same pathway. For instance, CDKs were obvious targets for inhibition in Patient #1 PDTOs (Table 1). However, when we attempted to use the information collected from the screening to identify additional CDK inhibitors with similar target profiles that would elicit expected responses, we were only partially successful (Fig. 2d and Supplementary Table 3). This could be due to different efficacies^30^, secondary targets, or other properties of the inhibitors. Therefore, our high-throughput approach allows not only to identify susceptible pathways but also to select the most effective agent within a class of molecules.

One important advantage of the mini-ring approach is the small number of cells needed. This allows testing samples as obtained from surgeries without the need for expansion in vitro or in vivo, a process that can lead to substantial divergence from the tumor of origin^31,43^. In our experience, the vast majority of solid tumor specimens does not adhere or grow in 2D, which limits the possibility of expansion in vitro. Moreover, take rates of patient-derived tumor cells in vivo can be highly variable^44^. We could only generate a limited number of PDXs from Patient #1’s cells over 5 months (3/12), whereas we could test 240 drugs in 5 days with a fraction of the cells. Therefore, our approach can be very effective to test patient samples that are recalcitrant to grow in vivo, reducing times and costs (Fig. 3d, e).

Another interesting application of PDTO screenings for precision medicine applications is in the rare disease space^45^. We could find several effective molecules against a carcinosarcoma of the ovary (Fig. 4b, Table 2). The rarity of this type of cancer, which accounts for only 1–4% of all ovarian tumors^37^, hinders the design of clinical trials to identify effective regimens. For instance, a clinical trial that demonstrated the efficacy of platinum agents in this setting run for ~20 years to enroll 136 patients^46^. As there is currently no standard, optimized first-line drug regimen for carcinosarcoma, therapy is usually modeled on other cancer types^47,48^. The ability to model rare tumors using PDTOs and perform robust screenings ex vivo offers an opportunity to identify drugs in a disease- and mechanism-agnostic manner, even for tumor types that are largely uncharacterized.

In conclusion, high-throughput drug screenings using PDTOs have many advantages and a real opportunity to be factored into therapeutic decisions. Our methodology can be a robust tool to standardize functional precision medicine efforts^3^, given its rapidity, with results potentially available a week after surgery, as well as ease of applicability to many different systems and drug screening protocols (Fig. 1, Supplementary Fig. 3 and 4). Although we used the mini-ring setup for drug screening purposes, the same methodology is suitable for studies aimed at characterizing organoids’ biological and functional properties with medium- to high-throughput. Complete automation (Figs. 3–4), flexibility to use different supports beside Matrigel (Fig. 2), and scalability to 384-well plates can further facilitate broader implementation of our mini-ring approach.

## Methods

### Cell lines and primary samples

Cell lines are cultured in their recommended medium in the presence of 10% fetal bovine serum (Life Technologies #10082-147) and 1% Antibiotic-Antimycotic (Gibco). DU145, PC3, PANC1, and HUTP4 were culture in Dulbecco’s modified Eagle’s medium (Life Technologies #1195-065). PAN03.27, MDA-MB-468, and MCF7 were cultured in RPMI (Life Technologies #22400-089). SK-NEP-1 was cultured in McCoy medium (ATCC #30-2007). All treatments are performed in serum-free medium (PrEGM, Lonza #CC-3166 or MammoCult, StemCell Technologies # 05620). All cell lines were obtained from and characterized by the UCLA Translational Oncology Research Laboratories.

### Primary samples

Primary ovarian cancer specimens were dissociated to single cells and cryopreserved or plated right after processing. In short, fresh tumor specimens or ascites samples were obtained from consenting patients (UCLA IRB). Solid tumor specimens are minced, then enzymatically digested with collagenase IV (200 U/ml). The resulting cell suspension is filtered through a 40 μm cell strainer.

For Patient #1 PDXs, 12 NSG mice were injected with 500 K cells in Matrigel on the flank (UCLA IACUC). Tumor growth was monitored over time. After about 5 months, three mice developed measurable tumors, collected after euthanasia. A portion was fixed and processed for histology, and the remaining tissue was dissociated to single cell and assayed following the same protocol adopted for the primary samples.

### Chemicals

Doxorubicin hydrochloride was purchased from Sigma (#44583). Staurosporine was purchased from Cell Signaling Technology (#9953S). BS-181 HCl (#S1572) was obtained from Selleckchem. Palbociclib (HY-50767A), AZD5438 (HY-10012), JNJ-7706621 (HY-10329), THZ1 (HY-80013A), and Flavopiridol (HY-10006) were purchased from MedChemExpress USA. All drugs were dissolved in dimethyl sulfoxide (DMSO). ReACp53 was synthesized by GL Biochem and prepared as described in Soragni et al.^17^ by resuspending in phosphate-buffered saline (PBS) at pH 8, filtering, and then diluting in media. For high-throughput screenings, we used a library of kinase inhibitors dissolved in DMSO available through the Molecular Screening Shared Resource at UCLA. Libraries are stored in the dark in a desiccator.

### 3D organoids seeding and treatment procedure

Single-cell suspensions (2K–15K/well, depending on the experiment as indicated in the text) were plated around the rim of the well of 96-well plates in a 3:4 mixture of PrEGM medium or Mammocult and Matrigel (BD Bioscience CB-40324) or Cultrex BME (Trevigen 3423-010-01). Cells in Matrigel or Cultrex BME are kept cold at all times and under continuous agitation, while generating rings. A short vortexing step is performed after every eight wells, together with a tip change. Warm PBS is added to all empty wells, if any. White plates (Corning #3610) were used for ATP assays, whereas black ones (Corning #3603) were used for caspase or calcein assays. Plates are incubated at 37 °C with 5% CO_2_ for 15 min to solidify the gel before addition of 100 µl of pre-warmed PrEGM or Mammocult to each well using an EpMotion (Eppendorf). Two days after seeding, medium is fully removed and replaced with fresh PrEGM or Mammocult containing the indicated drugs. The same procedure is repeated daily on two consecutive days. Twenty-four hours after the last treatments, media is removed and wells are washed with 100 µl of pre-warmed PBS. To prepare for downstream assays, organoids are then released from Matrigel by incubating at 37 °C for 40 min in 50 µl of 5 mg/mL dispase (Life Technologies #17105-041). All steps described above are performed with the EpMotion for all small-scale experiments (three-drug treatments) and medium is removed/added from the center of the wells.

### High-throughput drug screening

High-throughput drug screening experiments are performed using a Beckman Coulter Biomek FX integrated into a Thermo Spinnaker robotic system. In short, an intermediary dilution plate (Axygen P-96-450V-C-S) is filled with 100 µl/well of media and pre-warmed to 37 °C. Using sterile Beckman Coulter Biomek p50 tips, 1 µl of drug dissolved in DMSO at a 100 × concentration (12 µM and 100 µM stock concentrations) is transferred from the library compound plate to the intermediary media plate and thoroughly mixed. Next, the robot gently removed 100 µl of media from the matrigel/cell plate and disposes of it in an additional Axygen plate. As a last step, the robot transfers 100 µl from the intermediary plate (media + drug) to the matrigel/cell plate. The liquid handler is set up to hit the dead center of each well with no contact to the Matrigel mini-ring. Media is easily dispensed without touching or disrupting the Matrigel mini-ring. The total process time outside of the CO_2_ incubator is < 2 min per plate allowing the temperature to be controlled throughout. Only one set of disposable p50 tips is used for each plate. As indicated above, 24 h after the second treatment cells are released by incubating for 40 min in 50 µl of 5 mg/mL dispase (Life Technologies #17105-041) at 37 °C.

### Imaging

Plates are imaged daily for quality control purposes and to monitor organoid establishment and homogeneity of growth using a Celigo S Imaging Cell Cytometer (Nexcelom) in bright-field mode. For organoid number/size analysis, we gently shake plates for 2–5 min after release from Matrigel as described above, followed by a 2 min wait period to allow cells to settle on the bottom of the plates. Plates are then imaged in bright-field mode. We use the Celigo S Software for image segmentation and quantification of organoid number and area. Data are normalized to vehicle values and plotted with Prism 7.

### ATP assay

After the organoid release with dispase as indicated above, 75 µl of CellTiter-Glo 3D Reagent (Promega #G968B) is added to each well followed by 1 min of vigorous shaking. After a 30 min incubation at room temperature (RT) and an additional minute of shaking, luminescence is measured with a SpectraMax iD3 (Molecular Devices) over 500 ms of integration time. Data are normalized to vehicle and plotted, and EC_50_ values are calculated with Prism 7.

### High-throughput screening data analysis

For the high-throughput drug screening, DMSO and Staurosporine (1 µM) are used as negative and positive control, respectively. Cell viability values are normalized to vehicle (DMSO) and expressed as %. *Z*-scores are calculated as [(*Y*_drug X_) − (average *Y*_vehicle_)]/(average SD_vehicle_), where *Y* is either viability, organoid total number, or organoid area. The average SD_vehicle_ is a single value calculated across all three assay plates to better account for overall variability.

The three *Z*-scores, one for viability, one for organoid total number, and one for organoid area, are then averaged for each drug. This was performed separately for each patient.

Hits are determined following three criteria: (1) cell death shows concentration dependency, (2) residual cell viability at 1 µM is ≤ 25%, and (3) average *Z*-score ≤ − 5. For Patient #3, an average *Z*-score cutoff of − 1.5 was used. The different threshold was adopted due to heterogeneity in the vehicle SDs across subjects.

For Patient #2, partial hits are defined as drugs residual cell viability > 25% and ≤ (Staurosporine + 5%) at 1 µM.

### Caspase-3/7 and Hoechst assay

After dispase treatment, 100 µl of Nexcelom ViaStain™ Live Caspase-3/7 staining solution is added to each well. The staining solution consists of 2.5 µM Caspase reagent (Nexcelom #CSK-V0002) and 3 µg/ml Hoechst (Nexcelom #CS1-0128) in serum-free RPMI medium. Plates are incubated 37 °C/5% CO_2_ for 45 min and imaged with a Celigo S Imaging Cell Cytometer (Nexcelom). Data are normalized to vehicle values and plotted with Prism 7.

### Calcein-AM and Hoechst viability assay

For this assay, 100 µl of Calcein-AM/Hoechst/PI viability staining solution are added to each well containing the released organoids. The staining solution includes the Calcein-AM reagent (Nexcelom CS1 #0119; 1:2000 dilution), PI (Nexcelom #CS1-0116; 1:500 dilution), and Hoechst (Nexcelom #CS1-0126; 1:2500 dilution) in serum-free RPMI medium. Samples are incubated for 15 min at 37 °C with 5% CO_2_ before imaging with a Celigo S Imaging Cell Cytometer (Nexcelom). Data are normalized to vehicle values and plotted with Prism 7.

### Sample preparation for immunohistochemistry

Cells processed for fixation are seeded in 24-well plates to facilitate collection. Rings are washed with pre-warmed PBS, followed by 30min fixation at RT with 4% Formaldehyde EM-Grade (Electron Microscopy Science #15710). Samples are collected in a conical tube and centrifuged at 2000 × *g* for 10 min at 4 °C. Pellets are washed with PBS followed by a second spin. After discarding the supernatant, cells are mixed in 10 µl of HistoGel (Thermo Scientific #HG-40000-012). The mixture is shortly incubated on ice for 5 min to solidify the pellets before transferring to a histology cassette for standard embedding and sectioning.

### Immunohistochemistry

The slides are baked at 45 °C for 20 min and de-paraffinized in xylene followed by washes in ethanol and deionized water. Endogenous peroxidases are blocked with Peroxidazed-1 (Biocare Medical #PX968M) at RT for 5 min. Antigen retrieval is performed in a NxGEN Deloaking Chamber (Biocare Medical) using Diva Decloacker (Biocare Medical #DV2004LX) at 110 °C for 15 min for Ki-67/Caspase-3, PAX8 (Proteintech #10336-1-A), CDK7 (Sigma-Aldrich HPA007932), and p53 (Biocare Medical #CME298A) staining or using Borg Decloacker (Biocare Medical #BD1000 S-250) at 90 °C for 15 min for Anti-P Glycoprotein (Abcam #EPR10364-57) staining. For EGFR staining (Biocare Medical #ACI063 AK, CK), antigen retrieval is performed enzymatically with Carezyme III Pronase (Biocare Medical #PRT957) at 37 °C for 5 min. Blocking is performed at RT for 30 min with Background Punisher (Biocare Medical #BP947H) at RT for 15 min for the EGFR staining. Primary antibodies are diluted in Da Vinci Green Diluent (Biocare Medical #PD900L) for CDK7 (1:300), Anti-P Glycoprotein (1:300), p53 (1:200), and PAX8 (1:1000) incubated at 4 °C overnight, or Van Gogh Diluent (Biocare #PD902H) for EGFR (1:30) incubated at RT for 30 min. The combo Ki-67/Caspase-3 (Biocare Medical #PPM240DSAA) solution is pre-diluted and added to the sample for 60 min at RT. Secondary antibody staining is performed with Rabbit HRP-polymer (Biocare Medical #RMR622G) for the Anti-P Glycoprotein, p53, CDK7, and PAX8 staining, or with Mouse HRP-polymer (Biocare Medical #MM620G) for EGFR. MACH 2 double Stain 2 (Biocare Medical #MRCT525G) is used for Ki-67/Caspase-3 combinatorial staining. All secondary antibodies are incubated at RT for 30 min.

Chromogen development is performed with Betazoid DAB kit (Biocare Medical #BDB2004) for Anti-P Glycoprotein, p53, CDK7, EGFR, and Ki-67, or Warp Red Chromogen Kit (Biocare Medical #WR806) for Caspase-3. The reaction is quenched by dipping the slides in deionized water. Hematoxylin-1 (Thermo Scientific #7221) is used for counterstaining. The slides are mounted with Permount (Fisher Scientific #SP15-100). Images are acquired with a Revolve Upright and Inverted Microscope System (Echo Laboratories).

### Reporting summary

Further information on experimental design is available in the Nature Research Reporting Summary linked to this article.

## Supplementary information

Supplementary Material

Reporting Summary

## Data Availability

The datasets generated in this work are available from the corresponding author.
