# Supplementary material for: A simple high-throughput approach identifies actionable drug sensitivities in patient-derived tumor organoids
Source: Commun Biol. 2019 Feb 25;2:78. doi: 10.1038/s42003-019-0305-x (PMC6389967; doi:10.1038/s42003-019-0305-x)
Supplement: Supplementary file 1 — Supplementary Material [file 42003_2019_305_MOESM1_ESM.pdf]

Supplementary Figures

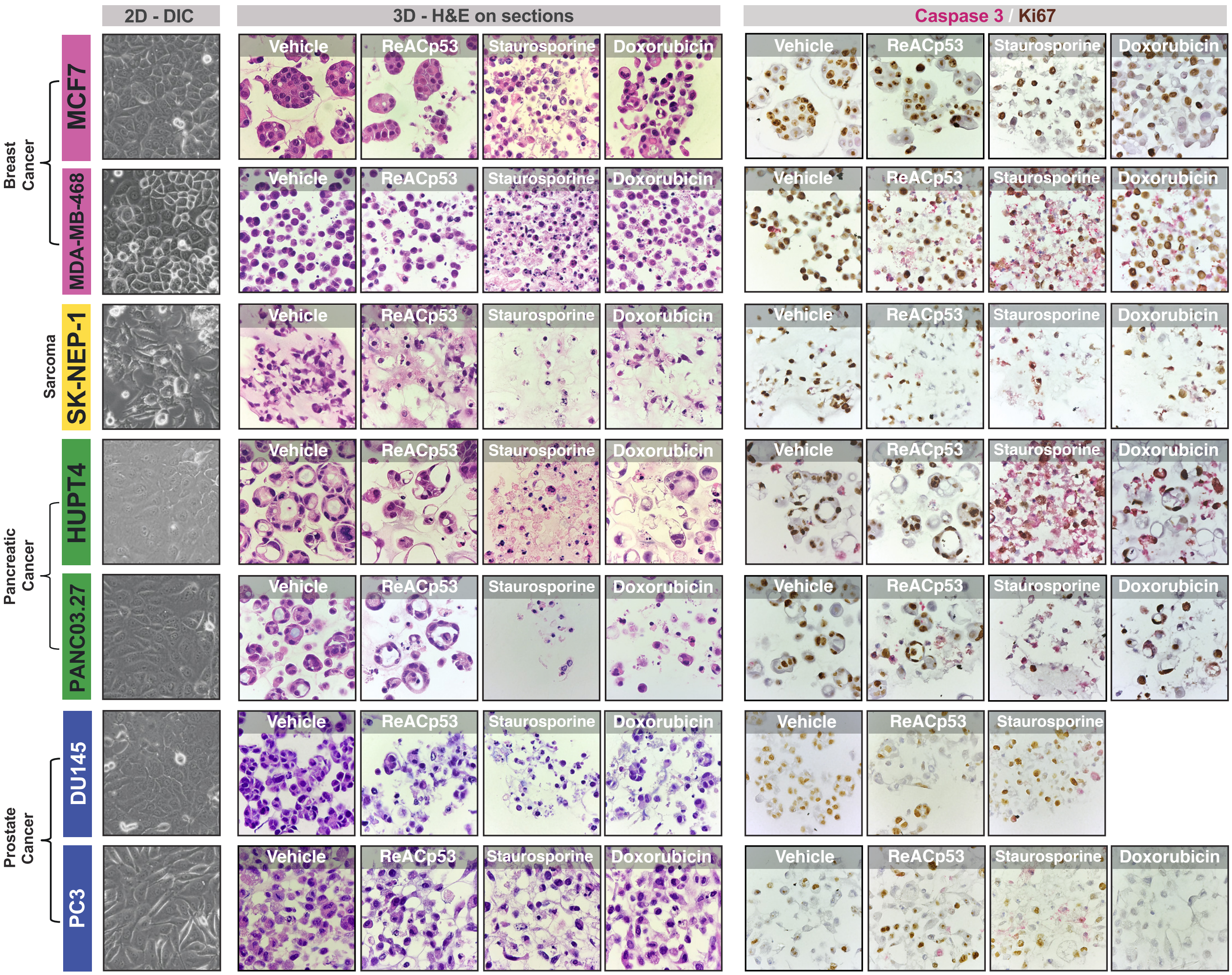

Supplementary Figure 1. Histology of 3D tumor models. Tumor cell lines used in this study grown in 3D processed for histology. The corresponding cells grown in 2D are shown on the left (40x magnification). On the right, H&E and Caspase/Ki67 staining on sections from embedded 3D tumor organoid samples (60x magnification).

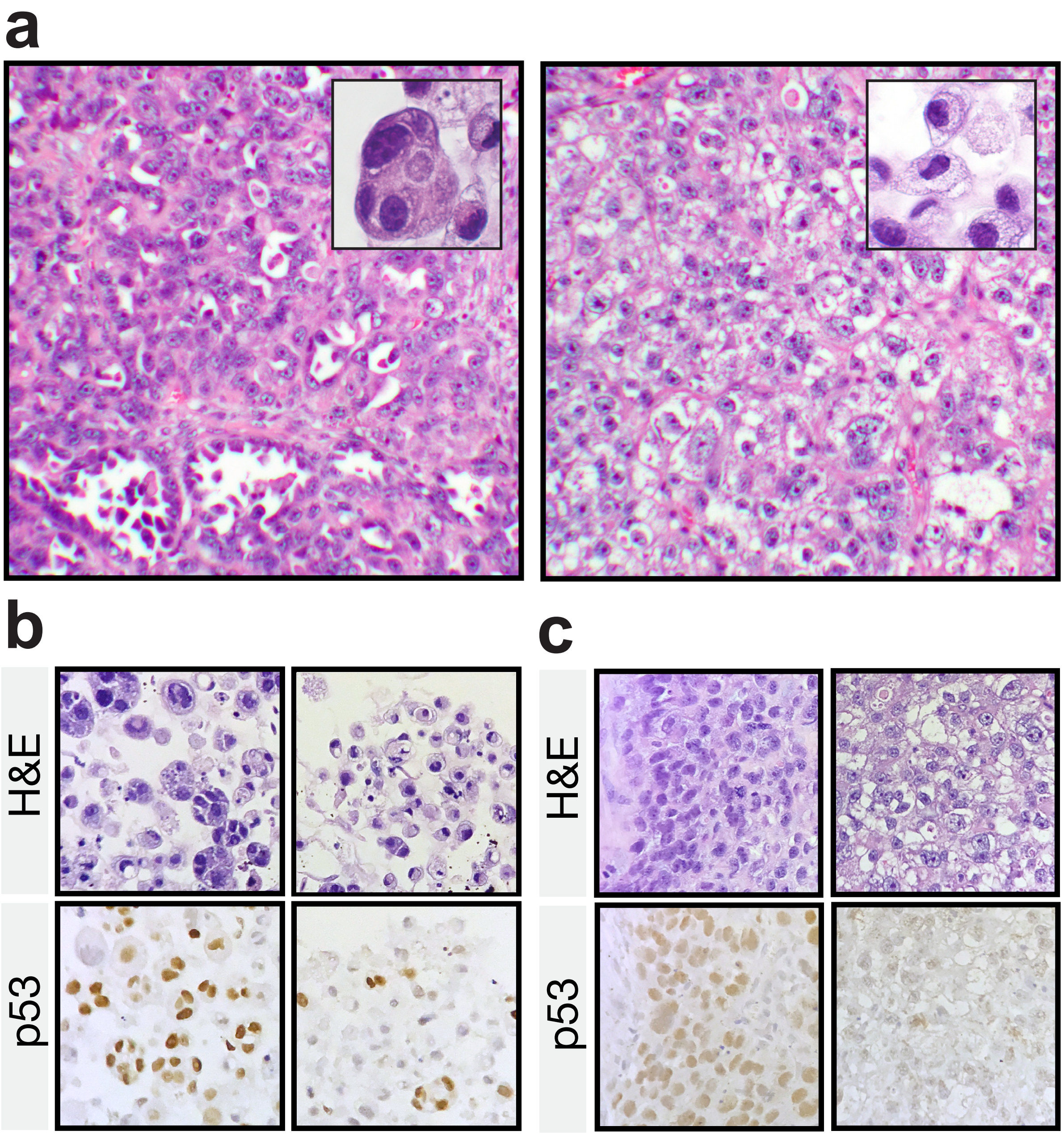

Supplementary Figure 2. Tissue sections of high-grade mixed carcinoma from Patient #1. (a) Different areas of the same tumor show distinct morphologies, with a high-grade serous component on the left and clear cell carcinoma aspects on the right. 10x magnification. The insets show a representative area of the tumor orgnanoids derived from the same sample. **(b)** p53 staining of Patient #1 organoids. The H&E image is representative of the overall area, but due to the limited thickness of the sample does not show the same exact organoids of the p53 staining. **(c)** p53 staining of Patient #1 primary tumor. In both **(b)** and **(c)** two populations of cells, a p53-positive (serous) and a p53-negative (clear cell), are evident.

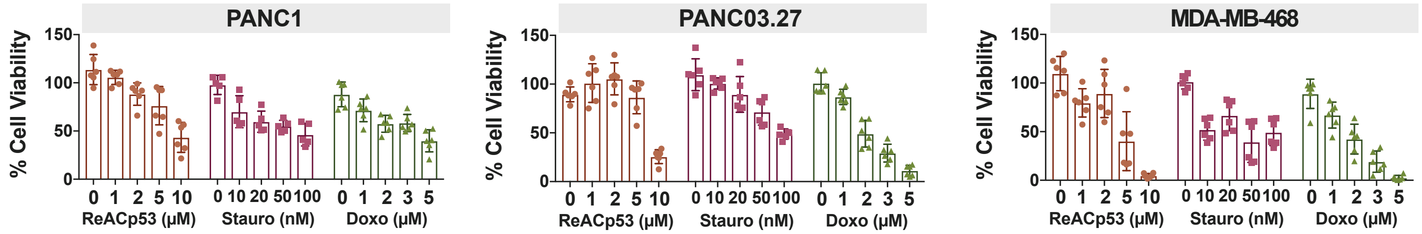

Supplementary Figure 3. ATP readout and EC_{50} values for three-drug assay. ATP quantification as measured by CellTiter-Glo 3D. Data from 2 independent experiments, n=3 for each are plotted. Error bars represent standard deviation; bars represent mean values.

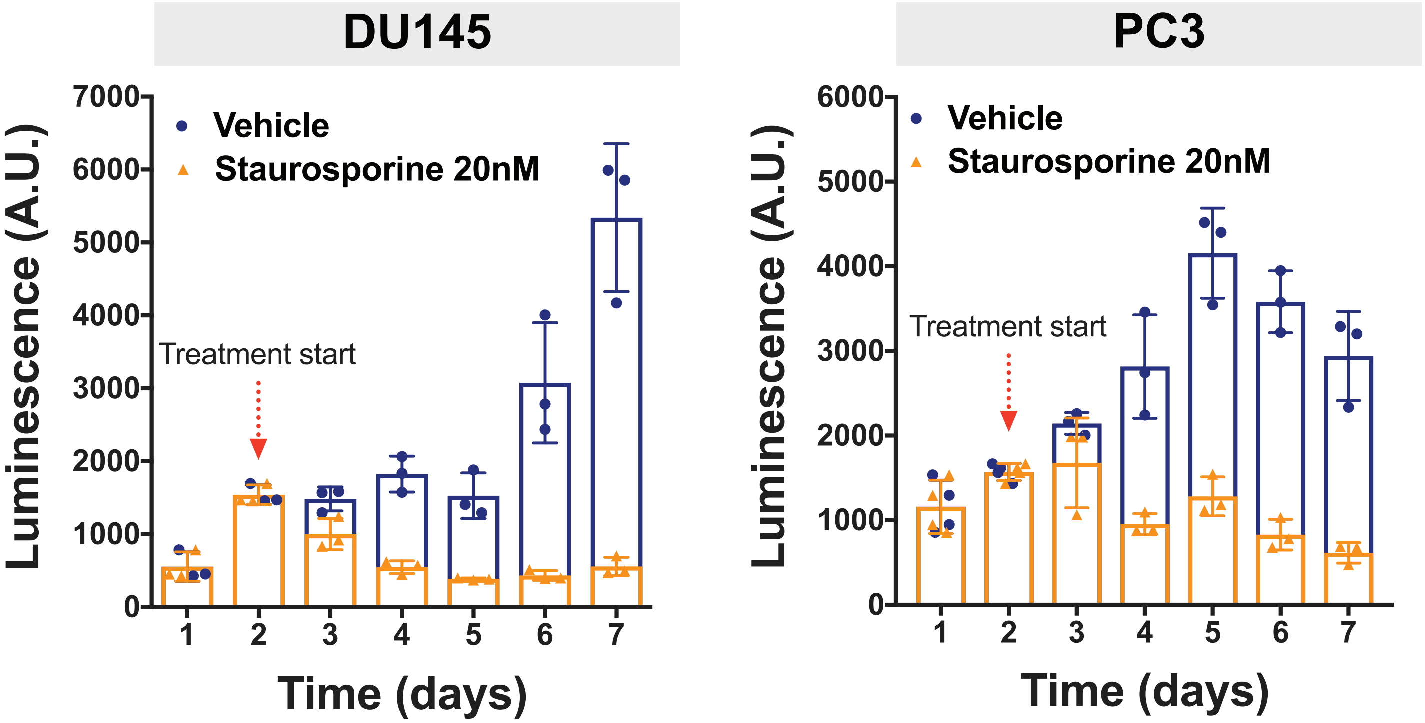

Supplementary Figure 4. Adaptability of miniring assay to different treatment schedules. ATP quantification as measured by CellTiter-Glo 3D of prostate cancer organoids treated for 5 consecutive days with either vehicle or 20 nM Staurosporine.

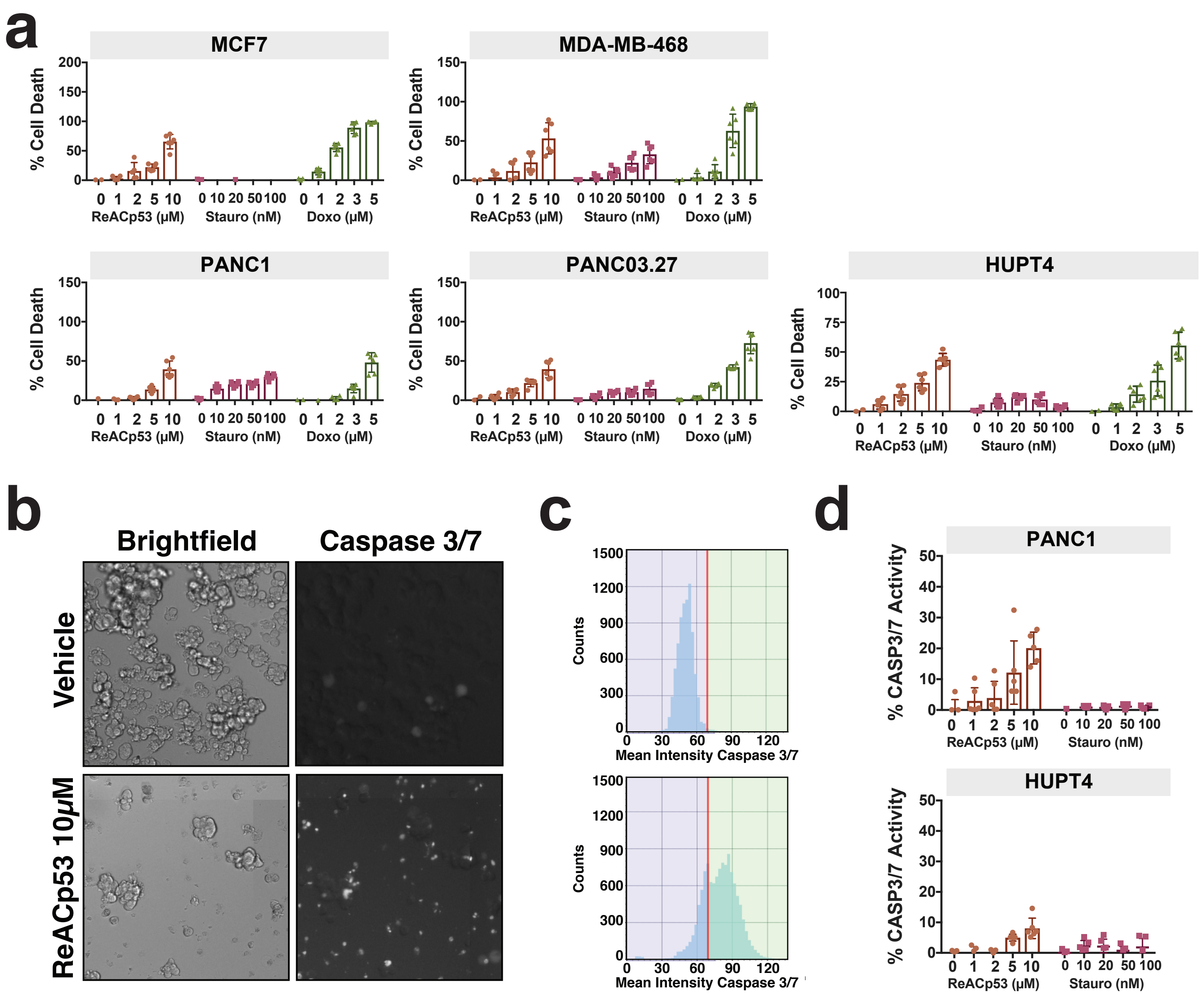

Supplementary Figure 5. Additional optimized readouts for mini-ring assay (a) Quantification of the calcein release / PI uptake experiment. Two independent experiments shown, n=3 for each. Error bars are standard deviation while bars represent mean values. **(b)** and **(c)** Example of outcome for the caspase 3/7 cleavage experiment. DU145 prostate cancer cells are shown. A substrate becomes fluorescent when cleaved by caspase 3 or 7. Treatment induces high levels of caspase activation. Histograms of fluorescence intensity are shown in **(c)**. **(d)** Quantification of active caspase 3/7 activity normalized to control. Doxorubicin has intrinsic fluorescence that masks the caspase signal hence was excluded from this analysis.

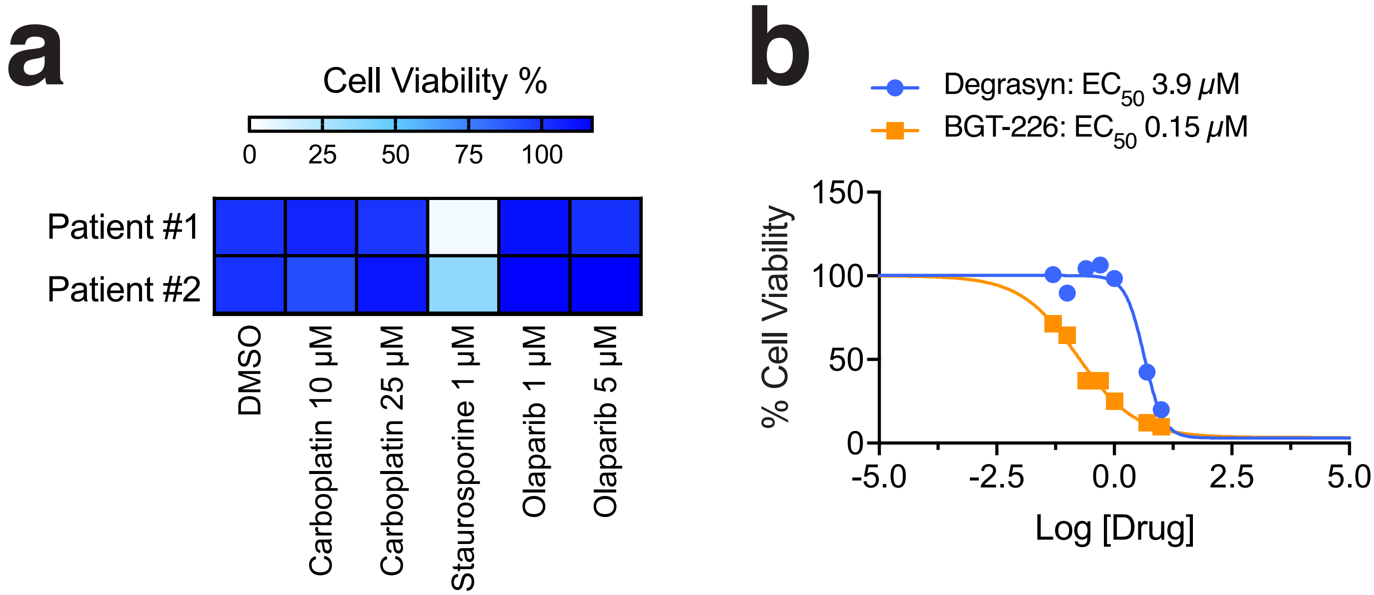

Supplementary Figure 6. Response of PDTOs to chemotherapy and screening validation. (a) PDTOs established from Patients #1 and #2 were also tested for response to Carboplatin and Olaparib. **(b)** Patient #2 PDTOs dose-response study for BGT-226 and Degrasyn. Data from two independent experiments was used to calculate EC_{50}.

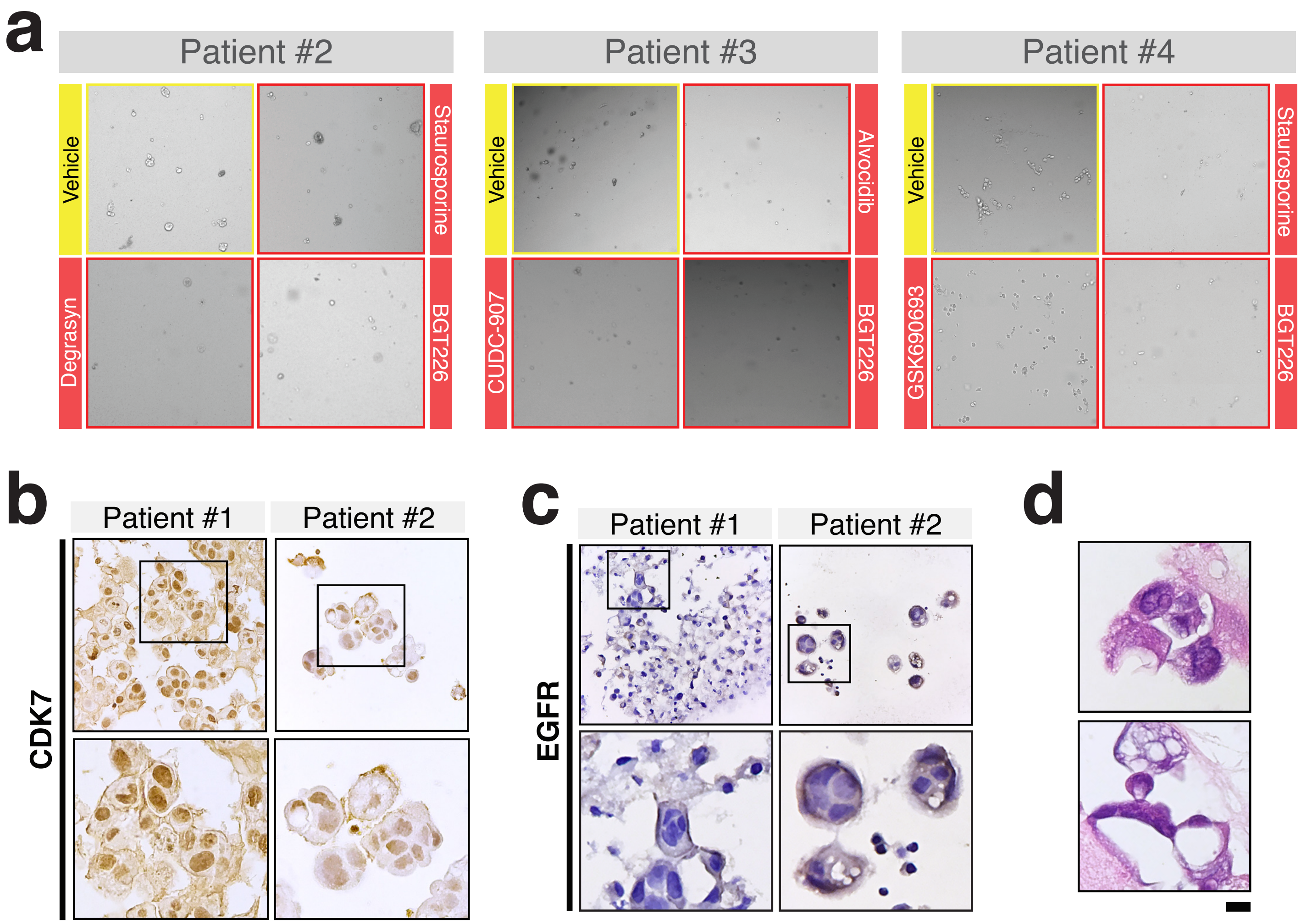

Supplementary Figure 7. Results and additional validation of PDTO kinase screening. (a) Representative images of post-treatment and post-dispase PDTOs. **(b)** CDK7 staining of PDTOs from Patient #1 and #2**.** Magnification: 40x. **(c)** Expression of EGFR in Patient #1 and Patient #2 PDTOs. Magnification: 40x. **(d)** Morphology of Patient #4 PDTOs, H&E staining of organoid sections. Scale bar: 10 μm.

Supplementary Tables

Supplementary Table 1. Characteristics of samples included in this study.

Stable Lines:

Specimen	Tumor Classification	Base Medium
**MCF7**	Invasive breast ductal carcinoma	RPMI
**MD-MBA-468**	Breast adenocarcinoma	RPMI
**PANC1**	Pancreatic ductal adenocarcinoma	DMEM
**PANC03.27**	Pancreatic adenocarcinoma	RPMI
**HUPT4**	Pancreatic adenocarcinoma	DMEM
**PC3**	Prostatic adenocarcinoma	DMEM
**DU145**	Prostatic carcinoma	DMEM
**SK-NEP-1**	Ewing sarcoma	McCoy

Clinical Specimens:

Specimen	Tumor Classification	Stage	Sample Type	Therapy
**Patient #1**	High-grade mixed type carcinoma with a high grade serous (40%) and a clear cell carcinoma (60%) component	IV	Ascites	None
**Patient #2**	High grade serous ovarian carcinoma	IVB	Ascites	Carboplatin / Taxol / Avastin
**Patient #3**	Carcinosarcoma of the ovary with malignant mixed Mullerian tumor	IIIC	Tumor	Carboplatin / Taxol
**Patient #4**	High grade serous carcinoma, primary peritoneal carcinoma	IIIC	Tumor	None

Supplementary Table 2. EC_{50} as calculated from ATP quantification data. All values are expressed in μM.

	ReACp53	Staurosporine	Doxorubicin
**SKNEP**	2.9	0.3	0.9
**MCF7**	9.9	0.1	12.0
**HUPT4**	7.4	0.3	1.5
**PANC1**	6.9	ND	5.7
**PANC03.27**	8.5	0.8	2.1
**MDA-MB-468**	2.5	ND	1.7

Supplementary Table 3. CDK inhibitors that did or did not induce cell death in >75% Patient #1's organoids. Targets and specificity of each is listed.

Patient did NOT respond to:

Targets:	CDK1	CDK2	CDK3	CDK4	CDK5	CDK6	CDK7	CDK9
**Roscovitine (Seliciclib)**		+			++			
**Milciclib**	+	++		++	+		++	
**PHA-793887**	++	++++		++	++++		++++	++
**PHA-767491**	+	+			+			+++
**BS-181 HCl**							+++	

Patient responded to:

Targets:	CDK1	CDK2	CDK3	CDK4	CDK5	CDK6	CDK7	CDK9
**Alvocidib****	+++	+++		+++		+++	+	
**R547**	++++	++++		++++				
**Dinaciclib (SCH727965)**	++++	++++			++++			++++
**AT7519**	++	++	+	++	+++	++	+	++++
**BMS-265246**	++++	++++		++				
**Flavopiridol HCl****	+++	+++		+++		+++	+	
**SNS-032 (BMS-387032)**	+	+++		+	+		++	++++

Tested for response:

Predicted	Observed	Targets:	CDK1	CDK2	CDK3	CDK4	CDK5	CDK6	CDK7	CDK9
Yellow	Yellow	Palbociclib HCl				++++		+++		
Red	Red	AZD5438	+++	++++						+++
Red	Yellow	JNJ-7706621	++++	++++	++	+		++		
Yellow	Red	THZ1							++++	
